# Postoperative Subdural Empyema Caused by Staphylococcus pseudintermedius: A Case Report

**DOI:** 10.7759/cureus.113467

**Published:** 2026-07-27

**Authors:** Abdel-Rahman Zakieh, Judy Teran, Subhashis Mitra

**Affiliations:** 1 Infectious Disease, Michigan State University, East Lansing, USA; 2 Internal Medicine, Michigan State University, East Lansing, USA

**Keywords:** canine exposure, case report, decompressive hemicraniectomy (dhc), diffusion restriction (dwi), methicillin-resistant staphylococcus pseudintermedius, purulent infection of the central nervous system, staphylococcus pseudintermedius, subdural empyema, subdural hematoma (sdh), zoonotic pathogen

## Abstract

*Staphylococcus pseudintermedius* is a coagulase-positive staphylococcus that colonizes the skin and mucosal surfaces of dogs. Although human infection remains uncommon, it is increasingly recognized as an emerging zoonotic cause of invasive disease. We report, to our knowledge, the first documented case of postoperative subdural empyema caused by methicillin-resistant *Staphylococcus pseudintermedius* (MRSP) in a 72-year-old man with household canine exposure. The patient initially underwent hemicraniectomy for evacuation of a traumatic subdural hematoma and subsequently re-presented with progressive neurologic decline requiring repeat neurosurgical intervention. Intraoperative findings revealed purulent material within the subdural space, and nine separate operative cultures yielded MRSP. Despite appropriate surgical source control, prolonged intravenous vancomycin therapy with therapeutic drug monitoring, and radiographic improvement, the patient ultimately died from complications of severe underlying neurologic injury. This case expands the recognized spectrum of invasive human *S. pseudintermedius* infection and highlights the importance of integrating clinical, radiographic, and microbiologic findings when evaluating postoperative intracranial fluid collections to facilitate timely diagnosis and management.

## Introduction

*Staphylococcus pseudintermedius* is a member of the *Staphylococcus intermedius* group (SIG) and is a common commensal organism in dogs and a known cause of canine skin and soft tissue infections and otitis [1]. Although historically considered a rare human pathogen, zoonotic transmission to humans is increasingly recognized, particularly among individuals with close canine exposure [2]. Reported human infections include skin and soft tissue infections, bacteremia, prosthetic device infections, otitis externa, and osteomyelitis [3-5]. Additionally, methicillin-resistant *S. pseudintermedius* (MRSP) is increasingly recognized as an emerging multidrug-resistant zoonotic pathogen [2]. Reported invasive infections more frequently occur in individuals with close canine exposure and are frequently associated with underlying comorbidities, immunocompromised states, or prosthetic material [1,2]. Central nervous system (CNS) infections caused by *S. pseudintermedius* are exceedingly rare, and to our knowledge, no cases of subdural empyema have previously been reported.

Subdural empyema is a life-threatening intracranial infection characterized by purulent collection within the potential space between the dura mater and arachnoid mater. Unlike the epidural space, the subdural space is less restrictive, resulting in a higher risk of wider spread of empyema and worse clinical outcomes [6]. It is a neurosurgical emergency requiring prompt neurosurgical evacuation in conjunction with targeted antimicrobial therapy [6]. Subdural empyema most often arises from contiguous spread of paranasal sinusitis or otitis and is most commonly caused by streptococci, *Staphylococcus aureus*, and anaerobic bacteria; however, unusual pathogens should be considered in patients with compatible clinical and radiographic findings [6].

Before the widespread adoption of molecular diagnostic methods, isolates belonging to the SIG were frequently identified broadly as *S. intermedius *[7]. In a reclassification study, Sasaki et al. reviewed 117 SIG isolates phenotypically identified as *S. intermedius* and demonstrated that most were, in fact, *S. pseudintermedius* [7]. Following recognition of *S. pseudintermedius* as a distinct species in 2005, the introduction of matrix-assisted laser desorption/ionization time-of-flight mass spectrometry (MALDI-TOF MS) and molecular sequencing revealed that many historical reports attributed to *S. intermedius* likely represented *S. pseudintermedius* [7]. This diagnostic limitation has likely contributed to historical underrecognition of the organism’s role in human infection. We report what is, to our knowledge, the first documented human case of postoperative subdural empyema caused by *Staphylococcus pseudintermedius* and the first involving methicillin-resistant *Staphylococcus pseudintermedius* (MRSP).

## Case presentation

**Figure 1:**
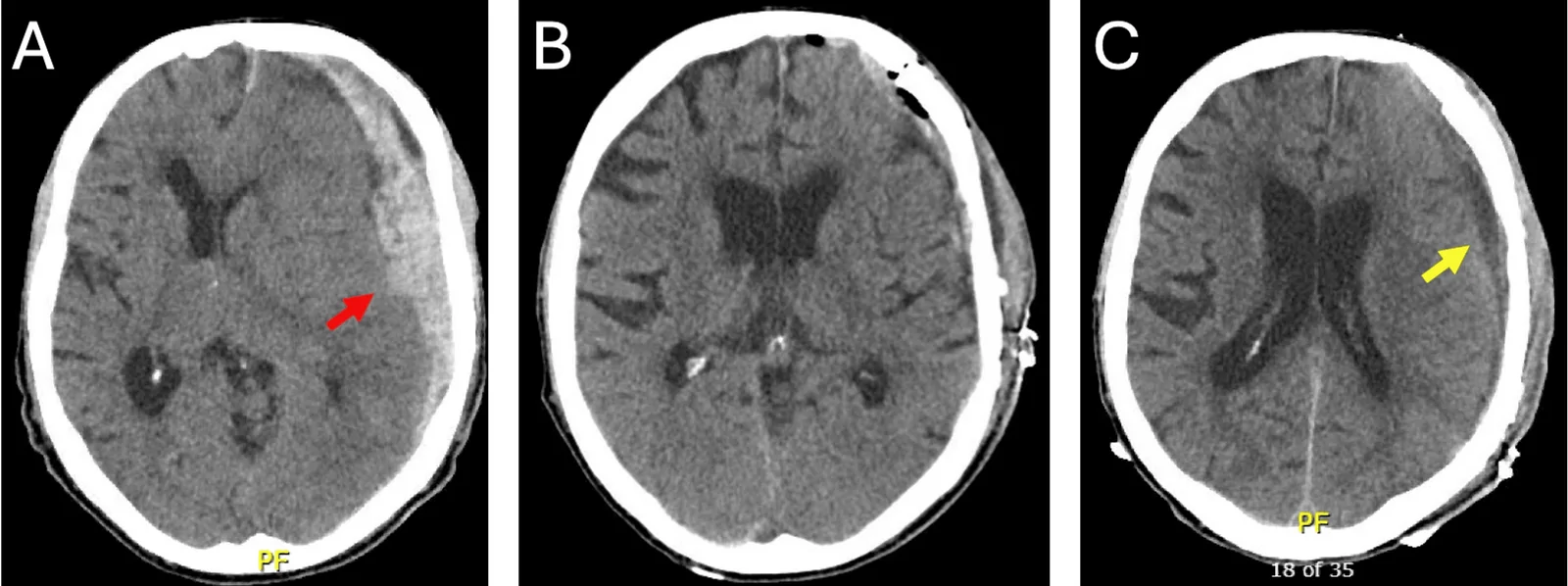
Serial CT imaging of the brain (A) Initial non-contrast CT of the head demonstrating a left-sided subdural hematoma (red arrow) with associated mass effect and midline shift following traumatic injury requiring hemicraniectomy. (B) Follow-up non-contrast CT of the head obtained on postoperative day 2 demonstrating interval evacuation with improvement in mass effect and expected postoperative changes. (C) Repeat non-contrast CT of the head obtained 10 days postoperatively demonstrating interval enlargement of the left subdural collection (yellow arrow) with mass effect confirmed intraoperatively to represent subdural empyema. CT: computed tomography

A 72-year-old man with a history of coronary artery disease, with a remote history of coronary artery bypass graft, type 2 diabetes mellitus, hypertension, hyperlipidemia, and chronic obstructive pulmonary disease, was initially admitted to the hospital with a traumatic left-sided subdural hematoma following a ground-level fall (Figure 1A) and underwent decompressive hemicraniectomy.

The patient did well postoperatively without persistent focal neurologic deficits and was discharged home on postoperative day 5. No postoperative antibiotics were administered beyond routine perioperative prophylaxis. Several days after discharge, he developed progressively worsening confusion, recurrent falls, and slurred speech, prompting presentation to the emergency department approximately one week later. There was no subjective or objective report of fever at home.

In the emergency department, the patient was hemodynamically stable and afebrile. On physical examination, he was able to follow commands and answer “yes” and “no” questions. The patient had no focal neurologic or cranial nerve deficits. Initial laboratory evaluation demonstrated stable anemia consistent with the patient’s baseline, without leukocytosis. Inflammatory markers and metabolic studies were largely unremarkable, with only mild elevation in procalcitonin and aspartate aminotransferase (AST) (Table 1). An isolated prolonged activated partial thromboplastin time (aPTT) was also noted on presentation (Table 1). Review of historical laboratory data demonstrated a persistently prolonged isolated aPTT dating back several years before presentation, with a near-normal prothrombin time (PT)/international normalized ratio (INR), suggesting a pre-existing coagulation abnormality.

**Table 1:** Initial laboratory evaluation on presentation ALT: alanine aminotransferase, AST: aspartate aminotransferase, BUN: blood urea nitrogen, INR: international normalized ratio, PT: prothrombin time, aPTT: activated partial thromboplastin time

Laboratory tests	Patient values	Reference ranges	Units
White blood cell count	9.4	4.0-12.0	×10^3^/uL
Hemoglobin	9.7	12.6-16.5	g/dL
Platelet count	378	150-400	×10^3^/uL
Neutrophils	84.3	49-81	%
Absolute neutrophil count	8.0	1.96-9.72	×10^3^/uL
Lymphocytes	9	14-41	%
Absolute lymphocyte count	0.80	0.56-4.92	×10^3^/uL
Sodium	139	136-146	mmol/L
Potassium	5.0	3.5-5.0	mmol/L
Chloride	106	98-108	mmol/L
Bicarbonate	23	20-31	mmol/L
Glucose	92	70-99	mg/dL
BUN	20	8-20	mg/dL
Creatinine	1.13	0.70-1.30	mg/dL
AST	39	0-33	U/L
ALT	31	10-49	U/L
Albumin	4.0	3.5-4.9	g/dL
C-reactive protein	<0.6	<0.6	mg/dL
Lactate	1.5	0.20-1.80	mmol/L
Procalcitonin	0.12	0.0-0.09	ng/mL
INR	1.2	0.9-1.2	-
PT	12.5	9.5-12.1	Seconds
aPTT	50.8	21-31	Seconds

**Figure 2:**
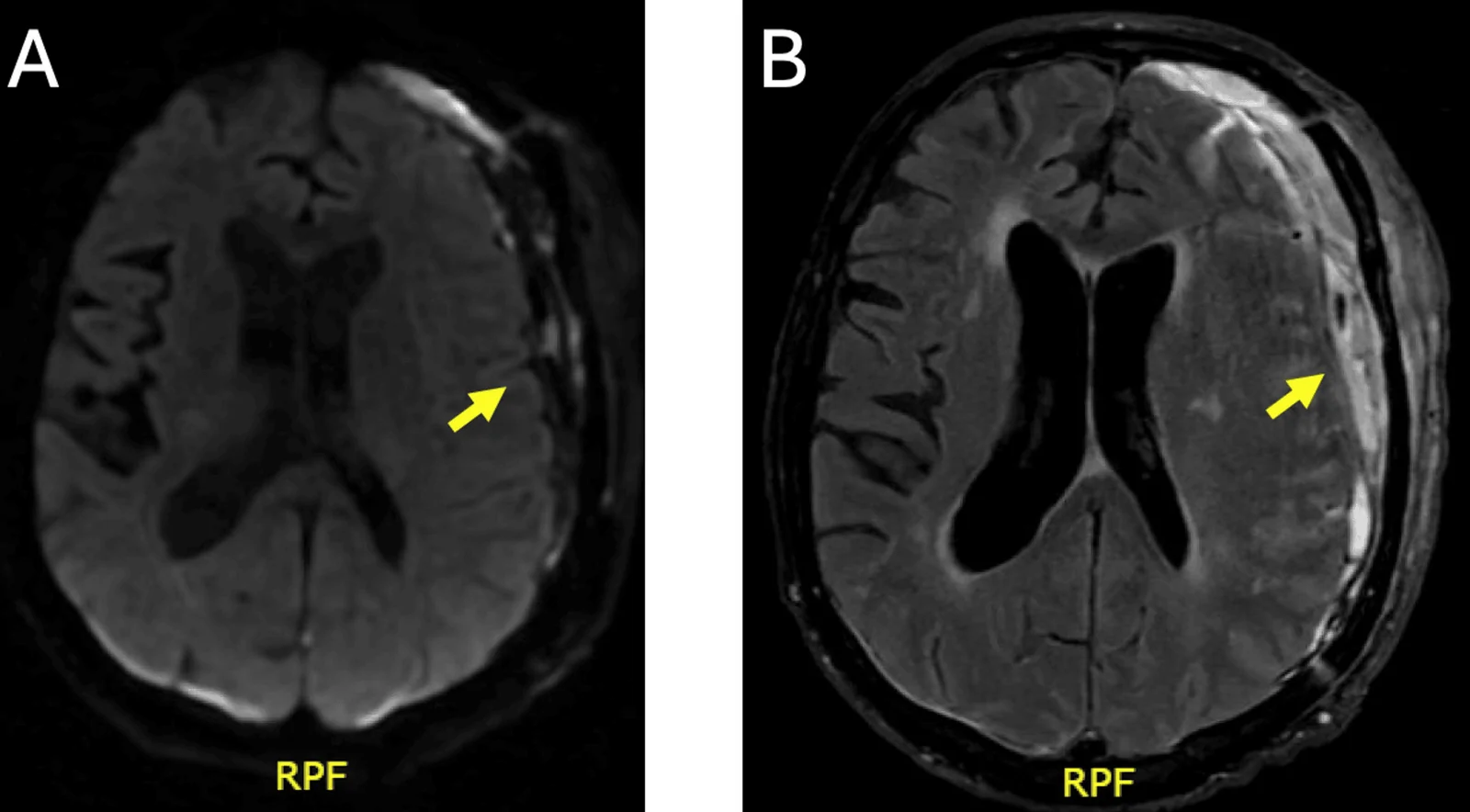
MRI of the brain demonstrating left-sided subdural empyema (A) Axial DWI sequence demonstrating marked diffusion restriction within the left subdural collection (yellow arrow). (B) Axial T2-weighted FLAIR image demonstrating extensive left hemispheric subdural fluid collection (yellow arrow) with adjacent edema and mass effect. DWI: diffusion-weighted imaging, FLAIR: fluid-attenuated inversion recovery, MRI: magnetic resonance imaging

Repeat imaging, including computed tomography (CT), CT angiography, and magnetic resonance imaging (MRI) of the brain (Figures 1, 2), demonstrated recurrence of the left subdural fluid collection. Due to initial concern for recurrent hemorrhage, left middle meningeal artery embolization was attempted; however, the vessel could not be visualized.

On hospital day 3, the patient developed an isolated fever of 100.7°F. Although postoperative hemorrhage remained in the differential diagnosis, the marked diffusion restriction within the subdural collection on diffusion-weighted MRI increased concern for subdural empyema. Subsequent neurosurgical intervention revealed purulent material within the subdural space, consistent with a subdural empyema.

Nine separate intraoperative specimens yielded growth of methicillin-resistant *Staphylococcus pseudintermedius* (MRSP), a coagulase-positive zoonotic staphylococcal species (Table 2). Operative specimens were inoculated onto routine aerobic (blood agar, chocolate agar, MacConkey agar, and Columbia CNA agar) and anaerobic (Brucella agar, laked blood with kanamycin and vancomycin (LKV) agar, and anaerobic CNA agar containing colistin-nalidixic acid) culture media. The organism was identified using matrix-assisted laser desorption/ionization time-of-flight mass spectrometry (MALDI-TOF MS) (Bruker Biotyper). Antimicrobial susceptibility testing was performed using BD Phoenix PMIC110 panel with interpretation according to Clinical and Laboratory Standards Institute (CLSI) M100 criteria. Methicillin resistance was determined by oxacillin susceptibility (MIC ≥ 1 μg/mL interpreted as resistant); mecA testing was not performed. The isolate demonstrated a multidrug-resistant phenotype, including resistance to oxacillin, clindamycin, erythromycin, and tetracycline (Table 3). Blood cultures remained negative despite multiple operative cultures yielding MRSP.

**Table 2:** Microbiological Studies and Culture Results a. Nine separate intraoperative specimens yielded MRSP on aerobic culture. AFB: acid-fast bacilli, MALDI-TOF MS: matrix-assisted laser desorption/ionization time-of-flight mass spectrometry, MRSP: methicillin-resistant *Staphylococcus pseudintermedius*, WBCs/HPF: white blood cells per high-power field

Specimen	Test	Findings
Blood	Blood cultures	No growth
Subdural operative specimen (n=9)	Gram stain	30-100 WBCs/HPF with few gram-positive cocci
Subdural operative specimen (n=9)	Aerobic culture	Growth of MRSP identified by MALDI-TOF MS^a^
Subdural operative specimen (n=9)	Anaerobic culture	No growth
Subdural operative specimen (n=9)	Fungal culture	No growth
Subdural operative specimen (n=9)	AFB smear/culture	No growth

**Table 3:** Antimicrobial susceptibility profile of MRSP isolated from a subdural operative specimen MRSP: methicillin-resistant *Staphylococcus pseudintermedius*, MIC: minimum inhibitory concentration

Antimicrobial agent	MIC (µg/mL)	Interpretation
Clindamycin	>4	Resistant
Daptomycin	≤0.5	Susceptible
Erythromycin	>4	Resistant
Linezolid	≤1	Susceptible
Minocycline	4	Susceptible
Oxacillin	>1	Resistant
Tetracycline	>8	Resistant
Vancomycin	≤0.5	Susceptible

Upon further discussion with the patient’s family, the social history was notable for close household canine exposure. The family reported no known direct contact between the dog and the patient’s craniectomy incision or other open wounds. The dog had been part of the household for several years and was reportedly up to date on routine veterinary care and vaccinations.

The patient completed a prolonged course of intravenous vancomycin, with dosing adjusted based on serial therapeutic drug monitoring, and was discharged to a rehabilitation facility. Therapeutic targets were maintained throughout the treatment course. Follow-up MRI performed approximately eight weeks after surgical drainage demonstrated interval reduction in the size of the left frontal subdural collection without radiographic evidence of recurrent empyema. His clinical course was complicated by readmission for three days, approximately one month postoperatively, for iatrogenic hypoglycemia and a mechanical fall without injury at the rehabilitation facility two weeks after discharge. Despite radiographic improvement of the empyema, he continued to experience progressive neurologic decline in the setting of significant underlying neurologic injury and multiple medical comorbidities. He ultimately died at the rehabilitation facility approximately eight weeks after surgical drainage.

## Discussion

This case represents, to our knowledge, the first documented report of subdural empyema caused by *Staphylococcus pseudintermedius *and the first involving methicillin-resistant *S. pseudintermedius* (MRSP). Beyond its novelty, this case highlights important diagnostic and therapeutic challenges associated with this emerging zoonotic pathogen. In addition to negative blood cultures and only minimal systemic inflammatory marker elevation, the diagnosis was further complicated by initial neuroimaging interpreted as postoperative hemorrhage rather than empyema. However, follow-up MRI demonstrated marked diffusion restriction within an enlarging subdural collection with adjacent cerebral edema and mass effect, prompting surgical exploration and microbiologic confirmation. Restricted diffusion on diffusion-weighted imaging reflects the high viscosity and cellularity of purulent material and is a characteristic MRI finding that helps distinguish between pyogenic intracranial infection and hemorrhage [6,8]. This case illustrates that postoperative *S. pseudintermedius* subdural empyema may present with limited systemic manifestations and underscores the importance of integrating clinical, radiographic, and microbiologic findings when evaluating postoperative intracranial fluid collections.

Although human infections caused by *S. pseudintermedius* remain uncommon, their true incidence is likely underestimated, particularly among invasive disease [1]. Unlike the predominantly skin, soft tissue, prosthetic device, and vascular access infections described in previous case series and reports, our patient developed an invasive postoperative intracranial infection, underscoring the potential of *S. pseudintermedius* to cause severe central nervous system disease [3-5]. Our case expands the recognized clinical spectrum of human *S. pseudintermedius* infection to include postoperative subdural empyema. Most reported infections had favorable clinical outcomes following source control and appropriate antimicrobial therapy, in contrast to our patient, whose postoperative intracranial infection occurred in the setting of severe underlying neurologic injury and ultimately resulted in death despite surgical drainage and prolonged targeted antimicrobial therapy.

The route of infection in our patient remains uncertain. Although postoperative inoculation with subsequent contiguous extension appears to be the most plausible mechanism given the temporal relationship to hemicraniectomy, other mechanisms cannot be excluded. Hematogenous dissemination appears less likely given the persistently negative blood cultures throughout the hospitalization and the absence of another identified infectious focus. While the patient had longstanding household canine exposure, there was no reported direct contact between the dog and the craniectomy incision or other open wounds. Furthermore, no canine cultures were obtained to evaluate for colonization or infection. Nevertheless, because *S. pseudintermedius* is a common commensal organism in healthy dogs, zoonotic acquisition remains biologically plausible [1,2,9].

The isolate remained susceptible to vancomycin, linezolid, and daptomycin. Intravenous vancomycin was selected based on in vitro susceptibility and because it remains a recommended first-line agent for serious methicillin-resistant staphylococcal infections, and therapeutic drug monitoring confirmed maintenance of target exposure throughout the treatment course. Although linezolid achieves superior cerebrospinal fluid penetration and may represent a reasonable alternative in selected cases, published clinical experience with MRSP central nervous system infections is extremely limited. Consistent with the management of bacterial subdural empyema, prompt surgical drainage was critical to microbiologic source control and favorable radiographic improvement. In this patient, follow-up neuroimaging demonstrated interval reduction of the empyema without radiographic evidence of recurrence, suggesting microbiologic response despite the patient’s ultimately poor outcome.

This case highlights several important clinical lessons. *Staphylococcus pseudintermedius *should be considered among potential postoperative pathogens in patients with canine exposure, particularly when conventional pathogens are not identified. Accurate species-level microbiologic identification using contemporary techniques such as MALDI-TOF is essential because historical misclassification has likely underestimated the burden of human infection. This case underscores the potentially severe clinical course of post-neurosurgical CNS infections, particularly subdural empyema in older adults with comorbidities. Finally, careful assessment of animal exposure, prompt surgical source control, and susceptibility-guided antimicrobial therapy remain essential components of management.

## Conclusions

*Staphylococcus pseudintermedius* is an underrecognized zoonotic pathogen capable of causing severe invasive human disease, including central nervous system infection. This case highlights the importance of species-level microbiologic identification, awareness of historical misclassification within the *Staphylococcus intermedius* group, and careful assessment of animal exposure when evaluating postoperative infections. Although prompt surgical drainage, accurate microbiologic diagnosis, and targeted antimicrobial therapy remain fundamental to management, this case illustrates that severe postoperative subdural empyema may carry a poor prognosis despite appropriate treatment, particularly in older patients with substantial neurologic injury and medical comorbidities. Whether canine exposure should influence perioperative risk assessment or infection prevention strategies remains unknown and represents an important area for future investigation.

## References

[1] Moses IB, Santos FF, Gales AC (2023). Human colonization and infection by Staphylococcus pseudintermedius: an emerging and underestimated zoonotic pathogen. Microorganisms.

[2] Guimarães L, Teixeira IM, da Silva IT (2023). Epidemiologic case investigation on the zoonotic transmission of Methicillin-resistant Staphylococcus pseudintermedius among dogs and their owners. J Infect Public Health.

[3] Somayaji R, Priyantha MA, Rubin JE, Church D (2016). Human infections due to Staphylococcus pseudintermedius, an emerging zoonosis of canine origin: report of 24 cases. Diagn Microbiol Infect Dis.

[4] Starlander G, Börjesson S, Grönlund-Andersson U, Tellgren-Roth C, Melhus A (2014). Cluster of infections caused by methicillin-resistant Staphylococcus pseudintermedius in humans in a tertiary hospital. J Clin Microbiol.

[5] Van Hoovels L, Vankeerberghen A, Boel A, Van Vaerenbergh K, De Beenhouwer H (2006). First case of Staphylococcus pseudintermedius infection in a human. J Clin Microbiol.

[6] Ziai WC, Lewin JJ 3rd (2008). Update in the diagnosis and management of central nervous system infections. Neurol Clin.

[7] Sasaki T, Kikuchi K, Tanaka Y, Takahashi N, Kamata S, Hiramatsu K (2007). Reclassification of phenotypically identified staphylococcus intermedius strains. J Clin Microbiol.

[8] Harapan BN, Wehn AC, Kuschel L (2026). Postoperative brain abscesses and infections after neurosurgery: clinical characteristics, risk profiles, microbiological findings, and longitudinal DWI and ADC MRI analysis. Brain Spine.

[9] Bannoehr J, Guardabassi L (2012). Staphylococcus pseudintermedius in the dog: taxonomy, diagnostics, ecology, epidemiology and pathogenicity. Vet Dermatol.

